# Exploring the 15-Minute City concept for the urban outskirts: a systematic literature review

**DOI:** 10.1186/s12544-025-00743-8

**Published:** 2025-10-17

**Authors:** Daniela Arias-Molinares, Karst Geurs, Anna Grigolon, Baran Ulak, David Duran-Rodas, Bartosz McCormick, Jelten Baguet, Charlotte van Vessem, Heythem Adjeroud, Linda Dörrzapf, Georgia Charalampidou, Ognjen Bobičić, Domokos Esztergár-Kiss

**Affiliations:** 1 Department of Civil Engineering and Management, University of Twente, Enschede, The Netherlands; 2 Chair of Urban Structure and Transport Planning, School of Engineering and Design, Technical University of Munich, Munich, Germany; 3 MPACT ASBL, Brussels, Belgium; 4 Vrije Universiteit Brussel, Mobilise Mobility and Logistics Research Group, House of Sustainable Transitions (HOST), Brussels, Belgium; 5 Univ Gustave Eiffel, Ecole des Ponts, LVMT, 77454 Marne-la-Vallée, France; 6 Research Unit MOVE, Institute of Spatial Planning, Technical University of Vienna, Vienna, Austria; 7 Institut für Verkehrswesen, Universität für Bodenkultur Wien, Vienna, Austria; 8 Department of Transport Technology and Economics, Faculty of Transportation Engineering and Vehicle Engineering, Budapest University of Technology and Economics, Budapest, Hungary

**Keywords:** 15-Minute City, Proximity, Shared mobility, Urban outskirts, Accessibility, Governance, Business models

## Abstract

The 15-Minute City concept has gained a lot of attention in research and planning, aiming to increase access to essential services by foot and bicycle and create more attractive and vibrant neighbourhoods. Most research has focused on exploring the concept in central urban areas, where in many European cities essential services are already within a 15-min walk or cycle. This paper presents a systematic literature review that syntheses existing knowledge on the 15-min city topic, identifies literature gaps and describes missing elements or “the key enablers” that allow the concept to expand beyond urban cores, into peripheral areas where car dependence is typically high. Based on our systematic literature review examining 87 papers, there are three well-established spatial elements-density, diversity, and design—that are central to achieve proximity. However, we also identify other three critical but underexplored elements: individual’s characteristics and needs, the role of digitalisation, and adapted governance and business models. These gaps limit the applicability of the 15mC across all contexts, particularly in urban outskirts. Our findings aim to inform planners and policymakers on the elements that need to be addressed for proximity-based planning to be effective across diverse urban contexts.

**Supplementary Information:**

The online version contains supplementary material available at 10.1186/s12544-025-00743-8.

## Introduction

The 15-Minute City (15mC) has gained traction as a key planning concept with almost 100 cities around the globe identified as having 15mC practices, which shows how planners and authorities are embracing it in their planned visions [88]. The ideas behind this 15mC concept, are not new, as it builds upon the foundations already defined by previous researchers [19, 50, 75] like the garden city concept, neighbourhood unit plan, post-modern urbanism, eco-urbanism, Polycentric City, Cervero’s 6Ds, Time Geography, Transit-Oriented Development, New Urbanism and Chrono-Urbanism (see Supplementary material for origins’ timeline). All these movements promoted compact and neighbourhood-oriented settlements, but the automobile revolution altered the urban structure by making it possible to live/work or conduct daily activities further away. Hence, urban sprawl became the norm and cities were challenged with traffic congestion, pollution and social injustice issues. Since then, urban planners have been trying to revive traditional urbanism, reducing car dependency and encouraging sustainable mobility [16]. The focus has shifted to ensuring that people can easily access essential services and destinations by active modes. Especially after the global COVID-19 pandemic and its severe lockdowns in which many governments imposed unprecedented movement restrictions and quarantine measures. Physical contact was reduced to minimum and public transit environments were identified as risk zones for contagions. Walking, cycling and micromobility gained popularity, allowing residents to undertake their daily activities while maintaining social distancing measures [35, 39]. Urban health, which stayed as a secondary concern, became a primary one and confirmed the efficacy of complete neighbourhoods during health emergencies, showing that car-dependent neighbourhoods and cities based on modernist ideas and principles are not resilient during adverse events. This is the context in which, the 15mC, originally introduced by Carlos Moreno in 2016, was revived by Paris Mayor Anne Hidalgo, who promoted it in her re-election campaign during 2020 as part of a COVID-19 recovery strategy.

From the theory of “chrono-urbanism” created by Carlos Moreno [65], the 15mC advocates “*for an urban set-up where locals are able to access all of their basic essentials at distances that would not take them more than 15 min by foot or by bicycle*” [66], p. 100). In its own essence, the concept focuses mainly on urban areas, as places where “proximate access to everyday resources is easily attainable via the efficient provision of pedestrian, cycling and transit infrastructure to a sufficiently large population of consumers that provide demand for the amenities offered by local shops and service providers” ([72], p. 1). However, less attention has been paid to exploring how the concept could be adapted for different contexts beyond its original setting: the urban core. There is evidence now, of great inequalities within and across cities in terms of accessibility metrics [14] and therefore, the 15mC definition must also adapt to different contexts, as cities are not homogenous. A global accessibility analysis of 10.000 cities was already presented to visualise these inequalities and measure how cities are doing in their ideal 15mC [14]. Accessibility varies considerably, with disparities often following a core-periphery pattern where city centres are better served than outlying areas. Hence, it may not be feasible or practical to apply the same concept for every urban environment, particularly due to concerns about service quality disparity, local population densities, different individual needs, and geographical differences.

There is a need to consider Moreno’s ideas with a context-specific mindset, to avoid its implementation as a “technocratic, magic fix that ignores the complexities and challenges of social life” ([80], p. 16). Moreno himself has started to consider less densely populated areas around the label of *30-min territories* or *happy proximities* [66], highlighting the role of public transport and other mobility services to increase accessibility to essential needs for longer distances. Regardless of the terminology used or the specific threshold set, the core idea remains the same: to implement proximity-centred planning of essential services, and an explicit move away from car-dependency, resulting in shorter-distance trips. The vast majority of 15mC practices around the world focus on urban cores, with limited implementation in peripheral and suburban areas [88]. These areas, which we refer to as urban outskirts, are understood as those mid-dense neighbourhoods in the near context of an urban area, which tend to have higher car dependency and stronger economic and functional relationships with the city centre. Unlike urban cores, urban outskirts typically face a combination of spatial and infrastructural constraints: dispersed land use patterns, limited density to support diverse services locally, and fragmented or insufficient active mobility infrastructure [62]. Public transport networks often have lower coverage and frequency, making it difficult to offer viable alternatives to car travel, especially at nighttime as found in Wang, et al. [91]. Socially, these areas also tend to host a more diverse range of household structures, economic vulnerabilities, and mobility needs that are often overlooked in uniform proximity-based approaches. These structural conditions create unique barriers to implementing proximity-based planning and call for more tailored strategies.

It remains unclear how the principles of proximity can be transferred to the urban outskirts with low- and mid-density neighbourhoods. Therefore, the objective of this paper is twofold: (1) to synthesise the existing knowledge on the 15-min city, and (2) to identify literature gaps and missing elements that hinder the application of the concept in urban outskirts. We try to answer the following question: what are the elements that need to be considered when trying to achieve 15-min neighbourhoods in the urban outskirts? How similar or different are these elements in an urban core versus an urban outskirts? Understanding these elements is essential, as it can support more inclusive and context-sensitive planning strategies in peri-urban areas. By shedding light on the unique factors shaping the applicability of the 15mC model beyond the urban core, this study can help policymakers design more appropriate interventions for low- and mid-density contexts (like service regulations, accessibility measures and equity-oriented experiments), rather than applying strategies developed for central areas without adaptation. The rest of the paper presents the methodology (Sect. 2), followed by the literature review results (Sect. 3), and concludes with the discussion and conclusions (Sect. 4).

## Methodology

**Fig. 1:**
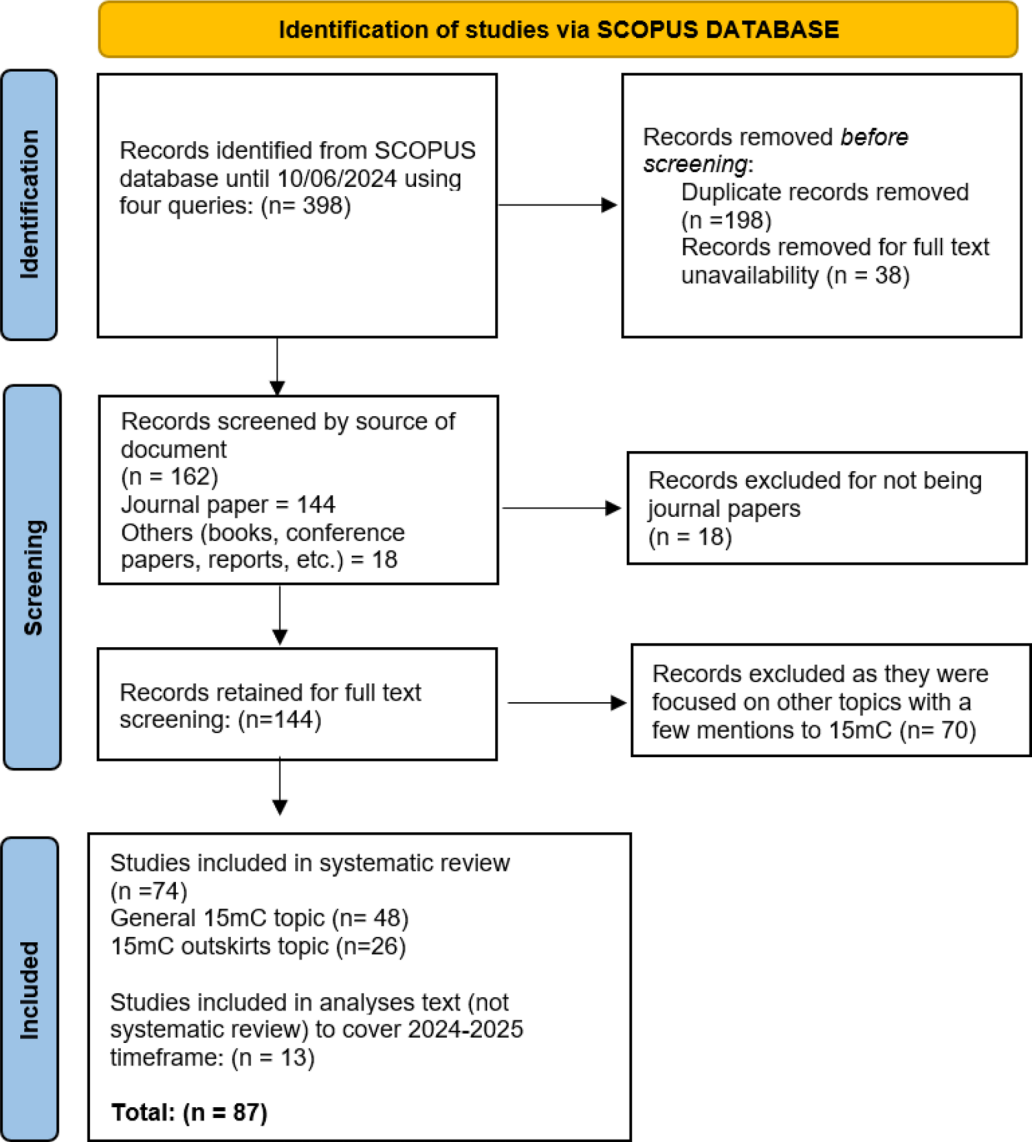
PRISMA flowchart for the selection of documents. Source: own elaboration

The review includes only academic scientific literature that has been found through the Scopus database. We decided to use Scopus exclusively, as we found a sufficient and robust literature base indexed in this database, and because it also enables efficient filtering to focus solely on peer-reviewed literature, excluding grey literature (compared to other databases like Google Scholar or WoS). We limited the search to peer-reviewed journal articles, as we found a sufficient volume of academic studies to support a synthesis. This focus ensured methodological transparency and allowed for an evidence-based understanding, distinct from the more narrative nature of grey literature. The documents reviewed were in English and in a final published stage. The review was conducted using the PRISMA (Preferred Reporting Items for Systematic Reviews and Meta-Analyses) guidelines [63]. A query to find any journal paper published that mentions the “15-min city” term in the title/abstract/keywords, as well as other topic keywords surrounding the research (accessibility, outskirts and shared mobility) was used (TITLE-ABS-KEY (“15-min city” OR “15 min city” OR “10-min city” OR “10 min city” OR “20-min city” OR “20 min city” OR “30-min city” OR “30 min city” OR “x-minute city” OR “x minute city” OR “x-minute region” OR “x minute region”) AND (LIMIT-TO (PUBSTAGE, “final”)). The search was conducted from March 1st until June 10th of 2024 obtaining 398 results. Many documents appeared repeatedly in the different queries’ results, so duplicates were discarded manually. For further document screening process, we only retained open-access records or those available via university account (144 records). Furthermore, a general screening of the documents was conducted, resulting in the discarding of some papers that only mentioned tangentially the 15mC concept. Finally, we coded and fully read 74 papers, which were categorised into two main groups: (1) general-documents that focus on the 15mC concept within urban city centres, and (2) outskirts-documents that either mentioned or delved into the 15mC implementation for outskirts or other settings beyond city centres. For each paper reviewed, we compiled a summary word document outlining its main findings and the specific topics addressed (density, diversity, design, individual’s needs, digitalisation or governance and business models). From the 74 documents revised, 48 fell in the general group and 26 in the outskirts one as seen in Fig. 1. We indexed the 74 papers (see Supplementary material for the authors’ tables), from 1 to 48 for the general literature and from 1 to 26 for the outskirts literature, based on the publication year (low number of index indicate older studies and high indicate more recent studies).

Finally, we have extended the search period to include publications from after June 2024 until August 2025. This update resulted in 13 additional relevant articles, bringing the total number of analysed documents to 87. While the main quantitative analyses and tables are based on the original set of 74 papers, the newly identified studies have been integrated into the qualitative synthesis and discussion where they offered novel insights or emerging trends. This approach maintains consistency with the original methodology while capturing recent developments in the field.

## Results

### Existing definitions of the 15-min city concept and their evolution

The most cited and highlighted definition of the 15-min city concept is the one offered by Carlos Moreno [66]. From the perspective of the idea called Flowers of Proximity, a 15mC is a “concept of urban planning that aims to create places where all essential services, such as work, education, healthcare, and recreation, are located within a 15-min walk or bike ride from each other” [15]. According to [9], 15-min cities are characterised by: easy accessibility, optimised location of services, socioeconomic equity, reduced use of private vehicles, and strong pedestrianisation. As we observe, definitions of the 15mC vary depending on the spatial scope of each research, as found also by Sepehri and Sharifi [79]. In the literature classified as general (focused in urban cores), the definitions mainly point to the 15-min threshold and the role of active mobility (walking and cycling). They often refer to goals related to reducing access to supermarkets/grocery stores and improving urban health. In contrast, the definitions found in the outskirts literature tend to be more reserved when it comes to setting a fixed time threshold and they even prefer to extend it to 20, 30, or even 45 min [10, 11, 17, 21, 54]. For example, [11] defined a 15mC as “a *city where most people have their employment and amenities accessible within a 30-min walk or public transport trip*”. In most cases, they stand by the broader terminologies of “x-minute city” or “x-minute territory” [51, 72]. Moreover, the outskirts definitions include public transport and shared mobility services in the mix (some even considered them as essential amenities) and the goals are mostly oriented to reduce car dependency in a regional area. Even though some variations are observed, the common elements remain the same in all the definitions: the emphasis on *proximity-centred planning* [66].

**Fig. 2:**
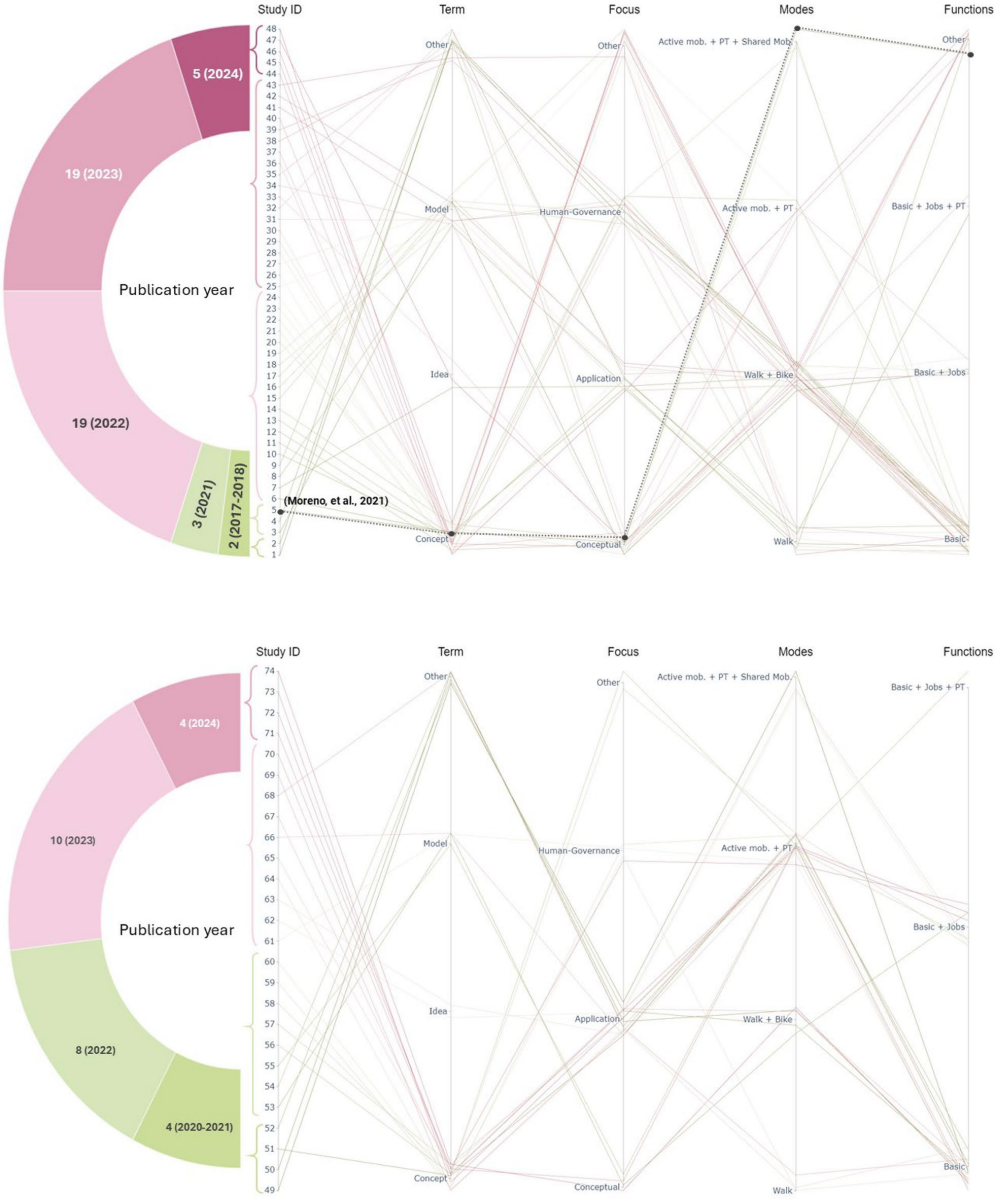
Evolution of general (48 studies) and outskirts (26) literature according to terms defining 15mC, focus, modes and amenities considered. Source: own elaboration. Basic amenities involves healthcare, education, leisure and commerce activities

Definitions show an evolution over time (see Fig. 2). It illustrates the year of publication, the term used by authors to define the 15mC, the focus of their research and the modes and functions (services) included in their analyses. In the case of the publication dates, we find that the documents in the general topic were published mostly during 2022 and 2023, but in the case of the outskirts topic, we see a latter pattern with most of them published during 2023. This pattern describes how in the beginning, as usually happens with all new concepts, the focus is on definition and application; while just some time after the topic has been established, new research begins to look further into gaps and overlooked topics, being one of those: the adaptation of the concept to the outskirts.

This is also noticed when delving into the focus of the studies, as we see that in the general literature, that there is a more homogeneously distributed interest in different topics. In the beginning (oldest studies), research mainly focused on defining and making critical analyses but more recently looked into diverse topics such as governance issues with 15mC [16], the inclusion of human and equity perspectives [6, 44, 46] or very specific topics such as the role of universities [7], park accessibility [47, 55, 95], technology [1–3, 56, 57], informal communities [24] or even mosques accessibility [43] in the 15mC. On the other hand, the outskirts literature, which emerges relatively later, focuses mainly on methodological approaches or practical studies that apply or test 15mC principles in different urban settings (urban core vs. urban outskirts) [32, 40].

Regarding the terms used to define 15mC, we see that at the beginning (oldest studies) many authors suggest different terms (“model”, “idea”, “planning policy”, “approach”, etc.) as it is a nascent and emerging topic, but then in both cases (general and outskirts literature), the most recent studies stand with the term “concept” when defining 15mC. Finally, in the case of the modes and functions included, the differences between the two piles of literature are very notorious. In the case of the general literature, most of the studies focus on walking and cycling and basic functions, while in the outskirts literature we see a clear pattern to include public transport in the mix showing the importance of this service for the outer peripheries [17, 21, 28, 72, 77, 93] and also including job functions [11, 22] or even public transport as a destination [4, 38, 56, 56, 57, 57, 69]. This evolution of the literature highlights the need to adapt the 15mC definition to different contexts, including public transport and shared mobility services that could help reduce car-dependency in lower density neighbourhoods.

### The three well-stablished necessary elements for 15mC: density, diversity and design.

The foundational concept of the 15-Minute City (15mC), as introduced by Moreno et al. [66], is built upon four core elements: density, diversity, proximity, and digitalisation. These elements closely align with Cervero’s well-known 3Ds—density, diversity, and design—(Cervero & Kockelman, 1997), offering a robust framework to understand how the built environment can facilitate short-distance, sustainable urban living. Our review reveals that the vast majority of academic studies primarily concentrate on these three elements, which consistently emerge in the literature as essential conditions for proximity-centred planning. In this section, we synthesise key findings related to what we refer to, as the three well-established basics for 15mC.

#### Density: a matter of scale

Most authors define the operational scale of 15-min city interventions using residential density, particularly metrics that consider only the urbanised area (excluding agricultural land, green areas, etc.). However, definitions of what constitutes a “city” versus a “neighbourhood” vary considerably across the literature. For instance, the Transect of Urbanism [23] suggests that mid-sized cities or urban outskirts typically exhibit residential densities ranging from 600 to 2.500 people/km2 and are primarily composed of residential zones with single-family homes on larger lots. These figures should be interpreted as indicative, as residential density thresholds are highly context-dependent and vary significantly across regions. Crucially, the level of density shapes the scale at which urban planning operates and, by extension, determines its primary focus.

Scale is one of the oft-mentioned critiques on the 15mC, as authors point to the risk of adopting a “one-size-fits-all” approach that fails to account for the unique characteristics of diverse urban environments [38]. Therefore, according to the area of intervention, planners should navigate through the different scales ranging from rural areas to city centres. For the focus of our research, two scales are relevant: urban city centres and their close periphery (suburban towns or urban outskirts). On the one hand, cities have an extraordinary quantity of amenities, from the more every-day and traditional to the more specialised and innovative ones, which coexist in relation to each other. It is the coexistence of different amenities -many of which are “rare” due to their location and distribution in the territory (e.g. museums, universities) and to the level of the demand—that produces the so-called “city effect” and defines the rank of cities [33]. Cities function as a system of neighbourhoods which are somehow self-sufficient for a certain set of services [23], and hierarchically dependent on higher-ranking services at the city level (e.g. Hospitals and Universities). Therefore, we can imagine a city as a network of neighbourhoods [10, 33, 50, 74].

When delving with the neighbourhood scale, on the other hand, the focus should be put on guaranteeing the use of essential services by all inhabitants through pedestrian paths, especially for essential ones like food and health. The quality of these pedestrian/cycling paths to reach the local essential amenities together with the quality of open spaces and urban layout become key at this level [12, 20, 32, 48, 50]. Therefore, the local scale focus is on the proximity of amenities within each neighbourhood, which means providing a wide array of services locally.

#### Diversity: available amenities

Once we determine the most fitting scale of the policy or intervention, the next step becomes to determine which amenities can be or should be offered at the local level and which at the city level. From our literature review, most of the studies use OpenStreetMap (OSM) as the main amenity data source, taking the same or similar categories. Some studies focused on specific amenities, like [11] which used only job locations and [20] which used only kindergartens.

**Fig. 3:**
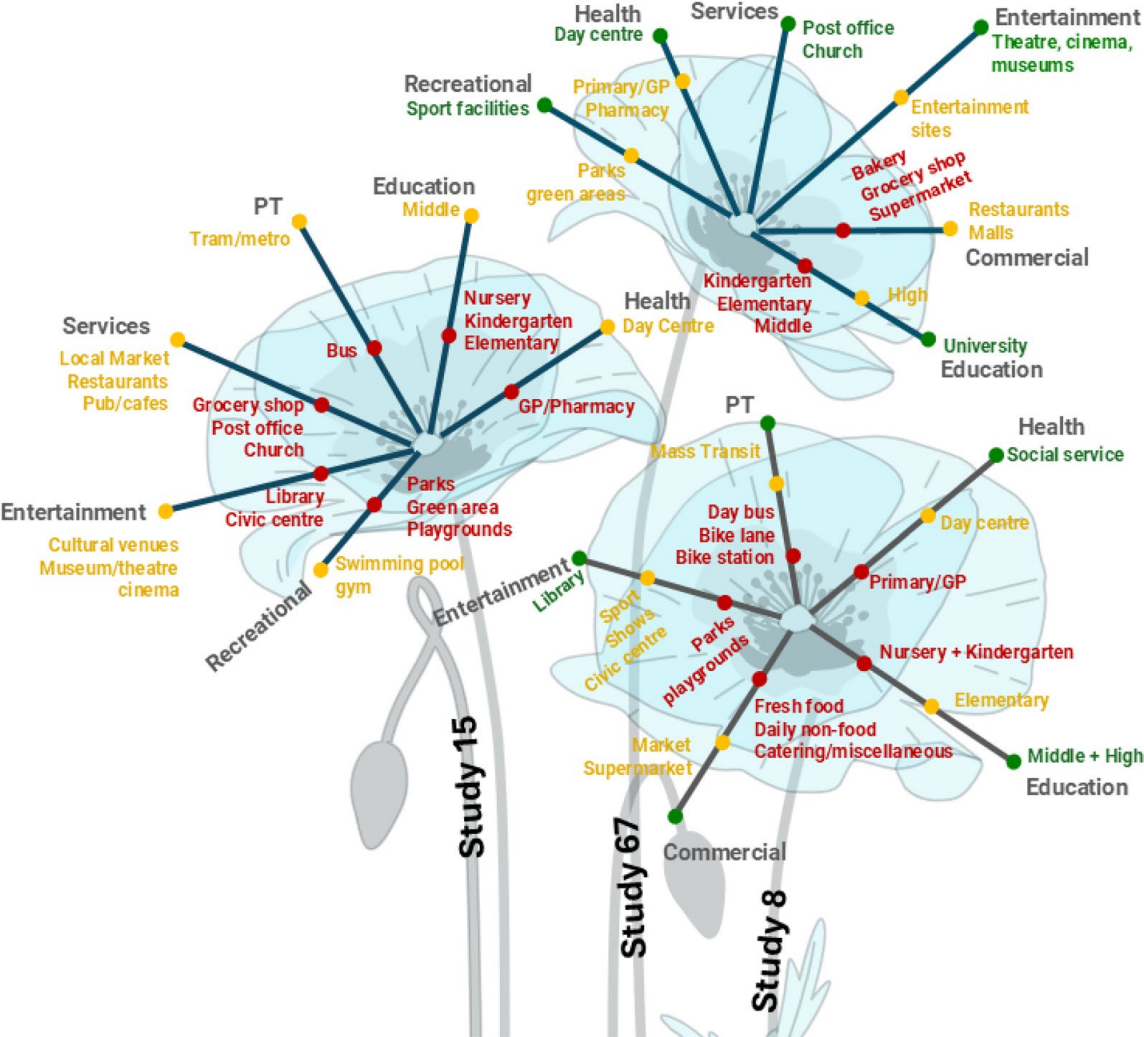
Flowers of proximity proposed by authors of study 8, 15 and 67. Source: own elaboration

Approaches and methodologies may vary, but most authors seemed to agree that some amenities require the accumulation of residents to be efficient/profitable [85]. Therefore, many experts recommend having an optimal (minimum) mix of amenities that fit the specific neighbourhood’s needs for a certain threshold [67, 68, 70]. One way to achieve this is by applying the 'Flowers of Proximity' approach, in which participants describe their desired level of proximity to essential services from their homes. The approach facilitates a co-creation process and allows for differences in needs and preferences to be explored [15, 83] (see Fig. 3). Flowers of proximity constitute a creative illustrative way of how people’s needs and preferences may vary from one neighbourhood to another. Some services are needed in close proximity (those in red in the figure), some others may be farther away (those in yellow) and others don’t necessarily need to be inside the neighbourhood (those in green).

From the literature review, the amenities categories used by authors vary in detail, with some authors even highlighting the “*subjective classification of essential services*” [26] (see Table 1), but generally agree on certain broad categories of services, such as education, healthcare, commerce (food-related in particular) and entertainment [17, 85]. We observe a variation in amenities considered by authors, with the prevalence of lower education or food services over higher education or specialised health centres, which reflects fundamental differences in planning and service delivery requirements. These later services require larger catchment areas, higher population densities, and more complex infrastructure. These conditions are often absent in low-density suburban contexts, making equitable access to for instance, hospitals/universities more difficult to achieve. This highlights the need for differentiated planning strategies within the 15-min city framework, adapted to the functional characteristics of each amenity type.

**Table 1:**
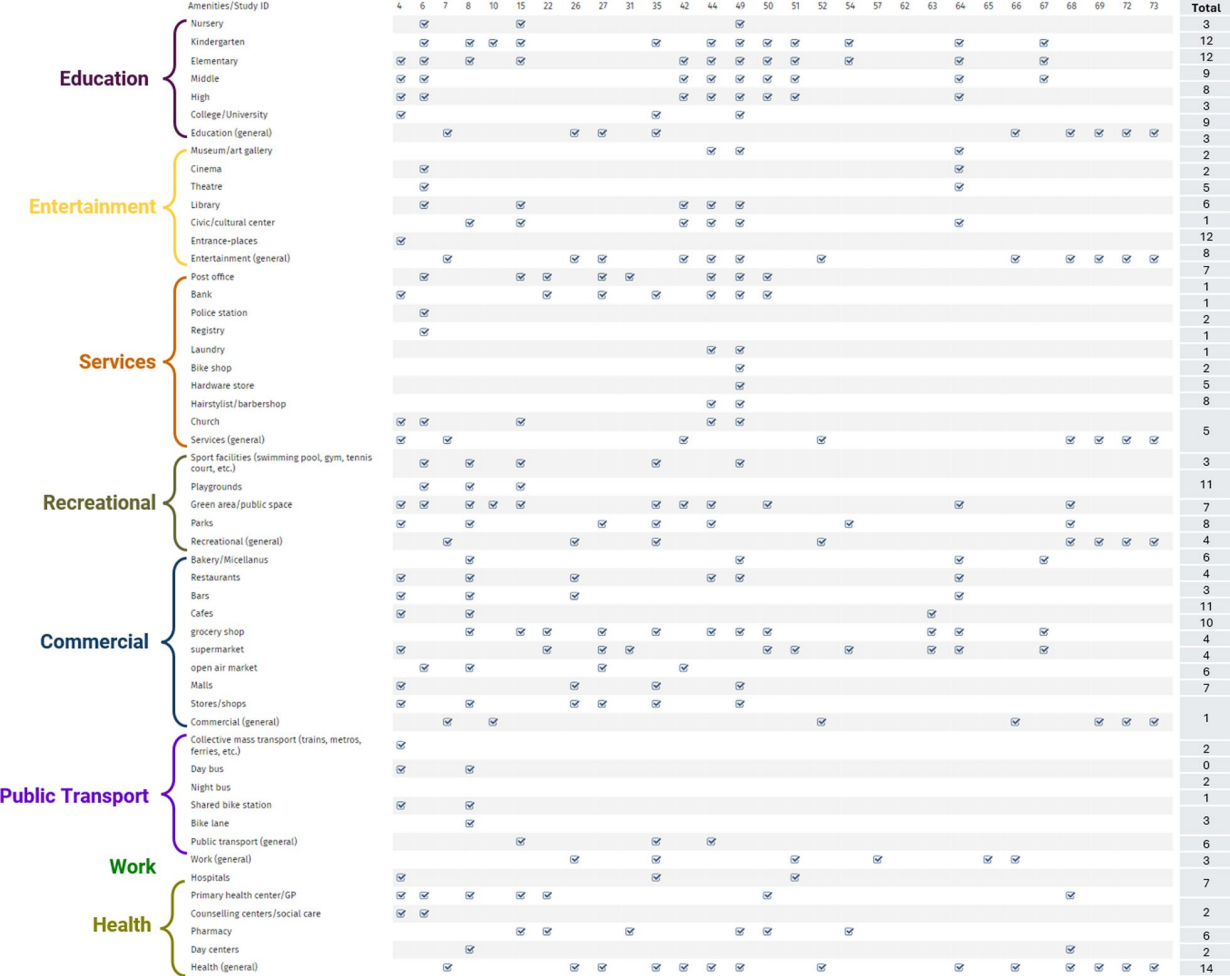
Amenities considered in the studies revised

Source: own elaboration

For the case of urban outskirts, some studies mention accessibility to public transport stops and infrastructure as relevant to reach jobs/education sites and other services at the metropolitan level [85, 86]. In the case of jobs, the literature is divided between authors that, on one hand, include jobs as an essential amenity [12, 34, 51, 76] and on the other hand, authors that considered jobs as a regional need [10, 21, 40, 58]. Authors that do not include jobs, argue that from a planning perspective, their access is more of a regional planning approach than a local one. From the few studies that analyse specifically work locations in the 15mC framework, we found a recent study by Li, et al. [53] which showed that many professionals in sectors like finance, education, health, and manufacturing in Melbourne commute over 20 min due to spatial mismatches between housing and centralised employment hubs shaped by agglomeration or logistical needs. In contrast, jobs in retail and hospitality are more spatially dispersed, making them more compatible with localised, 15-min city planning strategies. They proposed a three-pronged strategy—job decentralisation, improved suburban transit, and targeted housing infill near employment centres—emphasising that integrating land use and transport planning is essential to make the 15/20-min city model truly inclusive of work-related mobility.

#### Design: spatial setting for accessibility

After considering a neighbourhood’s scale (density) and its available amenities (diversity), design is what’s left: the spatial setting to allow built people to read these amenities. These component considers two main aspects: the built environment and connectivity.

Many of the reviewed studies examined built environment characteristics. The majority of them use the road/pedestrian network to characterise the cities in terms of walkability. For example, Gaglione et al., [33] considered slope, sidewalk width, cycling infrastructure, shaded paths, benches and other physical aspects to evaluate proximity to services on foot. Similarly, Di Marino et al., [22] addressed accessibility to job sites by walking using pedestrian network variables. In Birkenfeld et al., [10], researchers used two variables to measure the built environment: (1) the Walk Score method [25, 92] to address walkability levels, and (2) public transportation accessibility to job sites. Their study suggested that for some cities, especially car-oriented ones, applying the 15 and 30-min threshold could be challenging due to their existing urban layout (land use distribution and transportation infrastructure).

Similarly, connectivity have been shown to be highly relevant for the 15mC concept. Proximity is influenced by the spatial distribution and quantity of amenities in an area, but also by the quality, quantity and type of transportation options [42, 81]. Measures of access to public transport [11] and the number of jobs accessible within 30 min by different transport modes [31], illustrate how accessibility indicators can reflect the combined effect of mobility systems and service distribution.

Regarding the transport modes considered in the literature, Table 2 provides and overview. As shown, most studies include walking and cycling in their 15mC concept definitions. Some explicitly emphasise walking as central to the concept [4, 8, 20, 30, 33, 54, 94], while others recognise the essential role of public transport [11, 38, 72]. However, although 65% of studies (19 out of 29) reference walking and cycling in their conceptual definitions, the same proportion limits their analyses to walking alone.

**Table 2:** Transport modes considered in the studies revised

Study ID	Modes on the study’s 15mC definition					Modes included on the application of the study					Threshold used on study (min)						
	Only walking	Only cycling	Walking and cycling	Walking, cycling and Public Transport	Walking, cycling, Public Transport and Shared Mobility	Only walking	Only cycling	Walking and cycling	Walking, cycling and Public Transport	Walking, cycling, Public Transport and Shared Mobility	5	10	15	20	25	30	
Urban	4				x		x							x			
	6			x			x					x	x	x			
	7	x					x							x			
	8	x					x					x	x	x			
	10	x					x					x	x	x			
	15			x			x					x	x	x			
	22	x					x							x			
	26			x				x						x			
	27			x			x							x			
	31			x			x							x			
	35	x					x							x			
	42			x			x										
	44			x			x										
Outskirts	49			x						x					x		
	50			x			x							x			
	51			x					x					x			
	52			x			x							x			
	54			x			x						x	x	x		
	57				x						x						x
	62			x							x			x			
	63				x					x				x			
	64	x					x							x			
	65			x			x						x	x			
	66			x						x				x			x
	67			x						x							
	68	x					x							x			
	69			x			x							x			
	72			x					x					x		x	
	73			x										x		x	

(*) shared bikes considered (**)Literature review study considered micromobility trail paths

Source: own elaboration based on the 29 papers with applications on case studies

The role of public transport becomes particularly critical in peripheral or low-density urban areas where walking and cycling cannot meet all mobility needs. Fewer studies involve active mobility (walking and cycling) and public transport [10, 21, 64, 72] or even shared mobility services [11, 90]. For instance, in car-oriented cities such as Tempe (Arizona), Da Silva et al. [21] regard a 20-min public transport trip as acceptable within the city’s planning guidelines. In the Netherlands, Poorthuis & Zook [72] found that non-urban residents tend to rely on private cars to cover longer distances, even though their average trip durations approximate the 15-min ideal. They found both the city centres and non-urban areas maintain a rough average of 25 min per trip, but contrary to the ideal of the 15-min city, non-urban residents use personal cars to compensate for the longer distances that they need to cover.

In their study of Montreal, Birkenfeld et al. [10] showed that only 1.8% of households completed all daily activities within 15 min from home using active modes (walking, cycling, or public transport), and only 6% within 30 min. They highlight the structural limitations of the 15mC model in peripheries, stressing that proximity must be supplemented by robust and multimodal transport systems. Their work on the 30-min city underlines how access to essential opportunities in peripheral zones depends on service frequency, network integration, and cross-suburban transit -not just on the availability of modes. Accordingly, public transport should not be seen merely as a complement to active modes, but as a compensatory infrastructure that reduces spatial inequality and enables the inclusion of peripheries into proximity-based urbanism.

Both et al. [11] also highlight how cycling, particularly when supported by shared bike infrastructure near workplaces, has strong potential in extending 30-min access—demonstrating that mode combinations can help approximate 15mC principles even in less dense contexts.

### The overlooked elements that also enable 15mC: individual characteristics and needs, digitalisation and adapted governance and business models

Even though most studies on the 15mC focus predominantly on the physical elements discussed previously, our review also reveals other elements that, while less frequently addressed in the literature, are equally critical for the successful implementation of the concept. These overlooked elements are: individual characteristics and needs, digitalisation and adapted governance and business models. They constitute structural limitations of the current 15mC paradigm that apply across all contexts—especially in the urban outskirts.

#### Individual’s socioeconomic characteristics and needs

Individual’s socioeconomic characteristics and needs points to the key actors in 15mC: those called locals in Moreno’s 15mC definition. These are the citizens of the neighbourhood, which are the key stakeholders of policies the ones that build up the vision of the 15mC. In this component we delve into two aspects: (1) their socioeconomic characteristics and (2) their needs and preferences.

Socioeconomic characteristics are part of an individual’s life that are related to their social class and economic situation. These characteristics make up user profiles and allow planners to target specific target groups in their policies [44]. The most common examples of socioeconomic variables are gender, age and income, but also others that influence people’s mobility behaviour and certain spatial elements, such as the neighbourhood and housing type in which they live, the mobility modes they can afford, and the level of care/work that they can afford to outsource to others (such as bringing children to a daycare or hiring a grocery delivery service). These elements play an important role in shaping people’s needs in their neighbourhood, but so far, not much research has considered how these elements influence the needs of inhabitants of different neighbourhoods. Vehicle ownership is also considered in several studies. For example, Birkenfeld et al., [10] found that a household owning one or more vehicles was 78% less likely to be a 15-min household, and 87% less likely to be a 30-min household. Less common is to find documents that include education level and immigrant background [8, 38, 51].

With respect to gender, authors such as Aristizábal et al., [4] have emphasised the importance of promoting spatial justice and better opportunities for people, especially women, considering their diverse needs (caregivers) [5, 61, 82]. Even though some studies tangentially addressed gender-specific desired amenities, such as Guzman et al., [40], only one study by Soukhov, et al. [84] analyses the connection between 15mC and the “mobility of care” framework, emphasising access to care-related destinations—primarily used by women. Their study revealed that care-related accessibility in Hamilton is marked by spatial and socio-economic inequalities. Lower-income groups, who depend more on walkable care access, often live in better-served areas—but face growing displacement pressures from gentrification. Meanwhile, wealthier households tend to reside in less accessible neighbourhoods, highlighting challenges for equitable urban policy.

Generally, most studies on the topic of walkability and age have concluded that there are large differences in accessibility between city centres and suburban areas. The study by Rhoads et al., [78] focused on both older and younger people. Using data on the sidewalk network of Barcelona, they concluded that for both targets, access to vital services becomes limited not due to lack of services, but due to inadequate pedestrian infrastructure to reach them. A study in Santiago, which also focused on the access of older residents to grocery stores, showed that while the city centre was a promising accessible zone, the suburbs were still largely inaccessible by walking [89]. Similarly, a study on access to grocery stores by elderly population in Vancouver, showed a great accessibility of grocery stores by bike, but 15-min walking access was only possible in the central areas [45]. More recently, new research by Plaza-Herrera, et al. [71] in Barcelona found that while 81% of the overall population (across 62% of urban blocks) can access all services within a 15-min walk, only 42% of the elderly population meet this criterion-highlighting a major age-based accessibility gap.

Considering different target groups is key to promote inclusiveness, which is part of the concept of 15mC. This inclusiveness dimension needs indicators such as safety levels, people’s ability to move and affordability to be considered [19, 66]. The goal is to ensure access to essential services for all segments of society regardless of their abilities and socio-economic or cultural factors [6, 15]. Disadvantaged groups based on age, gender, race, migration background, language, income, education level, employment status, and disabilities need to be addressed. Therefore, both proximity and equity should be achieved, as a neighbourhood should be accessible not only in terms of proximity, but also in terms of inclusiveness and design for all. This is important to avoid gentrification (socio-spatial segregation caused by people leaving their neighbourhoods because they cannot afford to stay) and ghettoisation (isolation of lower income and vulnerable groups) as pointed out by Pozoukidou & Chatziyiannaki, [75]. To this end, the authors proposed the provision of equal opportunities for employment, education, lifelong learning, affordable housing, mobility options and financial resources, as also emphasised by Büttner et al., [15].

Besides population characteristics, individual needs and preferences must also be considered, as people may value different amenities and access conditions depending on their personal and socioeconomic situations. Characteristics such as age, gender, income, education, mobility capacity, and household structure influence how people perceive accessibility, the services they prioritise, and the modes they prefer or are able to use. For instance, while higher-income individuals may prioritise commercial and leisure services, lower-income groups often place greater emphasis on healthcare access, particularly among women with caregiving responsibilities [40]. Likewise, access preferences can vary significantly by mobility constraints—elderly or disabled populations may need shorter walking distances or better public transport, and car ownership strongly shapes travel mode preferences [41]. Recently, a study in Barcelona by Maciejewska, et al. [60] found that women, older adults, and individuals with lower education levels were more likely to adopt proximity-based travel behaviours, while younger and more educated people tend to travel farther in search of variety and specialised services. Cultural preferences, dispersed social ties, and ingrained habits also lead many residents to travel beyond their immediate neighbourhoods, even when living in compact, well-designed urban environments.

Understanding such diverse needs requires tools like surveys, focus groups, and interviews, which allow researchers to assess what residents want in their neighbourhoods, how they prefer to reach key amenities, and how much time they are willing or able to spend traveling. Despite this importance, only five of the reviewed studies conducted citizen surveys. Of these, only four were peer-reviewed academic articles [9, 24, 39, 40], while one was a governmental report [41]. However, none of the studies examined detailed amenity preferences specifically in the context of urban outskirts. Only Basbas et al. [9] included a sample from mid-sized cities (44%) and addressed preferences for broad categories of amenities, but the level of detail was limited, and only walking was considered—excluding cycling and public transport.

Other studies offered more granular data on specific amenity types. For example, Guzman et al. [40] asked participants in Bogotá to rank 24 specific amenities across six functional categories, revealing clear differences between income groups. However, this study did not assess acceptable travel times, reasons for preference, or preferred access modes. The report “Acceptable Accessibility” by KiM [41] provides a rich dataset on amenity preferences, acceptable travel times, and access modes across different population groups in the Netherlands. Findings showed that preferences for travel time, destination, and mode of transport differ significantly based on car ownership, urban density, and teleworking options. Yet, this remains grey literature (governmental report) and was not focused on suburban or peri-urban areas specifically.

Together, these findings underscore the importance of considering heterogeneity in users’ needs when applying 15mC principles to the urban outskirts. More research is needed to assess how various demographic groups define “accessibility” and what trade-offs they are willing to make, particularly in lower-density contexts.

#### The silent enabler: the role of digitalisation

Digitalisation was originally identified by Moreno et al. [66] as one of the four pillars of the 15-Minute City (15mC), yet it remains the least developed element in both conceptual discussions and empirical studies. While Moreno briefly mentioned digital technologies as enablers of reduced car dependency—such as through e-bikes, teleworking, telemedicine digital public services and online shopping—subsequent research has largely overlooked the transformative potential of digitalisation in enhancing accessibility, particularly in low-density or peripheral urban areas. Our review finds that digitalisation is rarely addressed in a systematic way, with only a few studies mentioning its role to improve planning practices while less attention has been paid to how it can extend access to opportunities without requiring physical proximity. This gap is notable given that these technologies could significantly reshape how residents in the urban outskirts experience and achieve the ideals of proximity and inclusion.

For instance, [1–3] explored how Digital Twins, IoT, and 6G-enabled networks could greatly enrich the spatial logic of the 15mC. By leveraging real-time urban sensor data and machine learning analyses, their study demonstrated how these technologies support adaptive, hyper-local planning, improve accessibility mapping, and respond dynamically to contextual needs. They also point to ways in which digital infrastructure can compensate for physical distance, especially n low-density settings, for example by offering telework/study or telemedicine instead of physical services/opportunities. In an similar study, Allam, et al. [2] positioned digitalisation as a foundational enabler for the functioning of density, diversity, and proximity within the 15mC framework. Rather than being peripheral, they argued that these digital tools are instrumental in modelling real-time urban dynamics, detecting gaps in service accessibility, and tailoring planning responses to local neighbourhood conditions. They highlight the relevance of smart sensors to monitor the usability of different public spaces such as parks, bicycle lanes, walking paths, car-free zones, etc., allowing the adoption of optimal strategies. Moreover, Khavarian‑Garmsir et al. (2023) highlights how digitalisation could help to enable citizen-facing dashboards, smart mapping applications, and livestreamed engagement platforms that promote more transparent and participatory governance at the neighbourhood level. Moreover, digitalisation supports Mobility as a Service (MaaS) frameworks and assists local businesses through data-informed decision-making, a combination particularly vital for extending accessibility and economic opportunity in low-density or peripheral neighbourhoods. More recently, Sepehri and Sharifi [79] delved into the potential of artificial intelligence (AI) to analyse large-scale urban data, simulate mobility scenarios, and monitor environmental conditions. They argue that integrating AI could support more adaptive, data-driven urban governance, yet current literature has yet to harness this potential to explore how it can be ethically and effectively aligned with the goals of accessibility, sustainability, and equity. Furthermore, Popescu and Nicolescu [73] emphasize the importance of integrating technologies such as Mobility-as-a-Service, shared micromobility, and intelligent transportation systems, as these can enhance accessibility, reduce emissions, and support well-being when fully embedded into the 15-min city model, rather than treated as mere add-ons.

Despite its limited treatment in the literature—with only five out of 74 papers revised describing the role of digitalisation in the 15mC—this element has emerged as a crucial gap and a silent but powerful enabler of the 15mC.

#### Adapted governance and business models

The last overlooked and critical element to enable 15mC is to consider different or adapted governance and business models. When analysing the 15mC topic literature, we found governance to be a uncommon topic. Authors mentioned briefly three aspects related to governance: policies, citizen participation and equity-oriented regulation. Regarding policies, Feng, et al. [29] found that simply promoting walkability is insufficient in the urban outskirts and policymakers should prioritise adding more facilities in targeted locations and consider promoting cycling as a complementary mode of transport. In the report ‘Mapping of 15-Minute City Practices’ [16] analysed 414 15mC practices in 98 cities around the world. They found that most of the cities focus on mobility and public space. The best practices mentioned were Barcelona’s Superblocks, Paris’s multi-purpose buildings, and Portland’s neighbourhood greenways. Successful examples of good governance include decentralised decision-making, district-level budget allocations for participatory workshops, and direct citizen participation.

Similarly, Gower & Grodach, [37] reviewed the planning documents of 33 cities worldwide to explore how the concept of 15-min neighbourhoods has been implemented. Their results showed that only two cities (Portland and Eugene) included specific, measurable benchmarks in their policy documents. The rest of the planning documents lacked measurable policy benchmarks, and no statutory weight was found, weakening planners’ ability to commit to the concept without clear understanding of what is expected to be delivered. The authors showed that many of the cities used the concept in their general guidelines (as a city-branding device) but do not specify how they are going to achieve this through policy, law and specific measures. Lu and Diab [59] critically showed how different interpretations of the 15mC concept—across cities in the United States, Canada, and Australia—resulted in divergent policies and thus outcomes depending on time thresholds, destination types, and local spatial contexts. On recent research, Caprotti et al. [18] argue that the governance of the 15-min city often reflects post-political tendencies, where technocratic approaches and elite-driven planning risk deepening inequalities and public mistrust. They recommend participatory and context-sensitive planning processes that engage with local socio-spatial realities and acknowledge dissent as a legitimate part of democratic urban governance. They caution that without such recalibration, the 15mC may serve more as a superficial branding exercise or a “neoliberal urban fix” than as a transformative framework for inclusive and sustainable urban development.

When trying to achieve higher citizen participation, authors recommend that citizens should be surveyed and engaged in the design and implementation of their neighbourhoods [74]. Collaboration should always be sought not only through digital means, but also through public meetings. Self-organised, bottom-up communities should be encouraged and facilitated, by providing spaces and equipment to allow meetings and interaction to happen. This participation can be incentivised, as is practiced in contemporary citizen science, with either non-monetary or monetary rewards in order to overcome the usual challenges associated with attracting adequate participation [74]. According to these authors, these workshops should help planners to achieve a governance plan for the neighbourhood towards 15mC. The governance plan should include at least: 1. Vision and objectives of the 15-min neighbourhood, 2. Key facilities and infrastructure to achieve the vision along with standards of operation, 3. Organisational structure roles, responsibilities of the multi-stakeholder team, 4. Procedures through which citizens and businesses will meet and work together for the vision, 5. Legal and ethical code for each stakeholder defining rights and obligations, and 6. a method for assessing the performance and impact of the 15-min vision on social, economic and environmental aspects [74].

Many authors have also highlighted the importance of coordination between different levels of administrations and authorities [16, 36]. Streams of investments directed toward implementing 15mC in the main central city could lead to increased inequalities between the main city centre and the immediate suburbs. Without sufficient coordination with regional actors, core investments fuelling the 15mC could risk a decline in public transport infrastructure investments. If public transport is not sufficient and widespread throughout the city and its suburban borders, there is a risk that commuters may resort to car use to cover longer distances.

Municipalities have a big role in this coordination as the link between local stakeholders and metropolitan authorities. Consequently, recent research done by Brown & Howell, [13] has also shown how municipalities can include equity-based requirements in tenders for shared mobility and micromobility operators, including:

- Policy Development: stating that cities should establish clear equity policies that target vulnerable groups, focusing on affordability, accessibility (including non-smartphone access), and geographic distribution to underserved areas.
- Equity in Implementation: governance frameworks should mandate specific features, like cash payment options and adaptive vehicles, which are critical to expanding access to a broader populations.
- Evaluation: reporting needs to include evaluation mechanisms to ensure compliance and assess whether equity goals are being met. This includes requiring operators to report usage data disaggregated by income, race, gender, and neighbourhood.
- Incentivising compliance: operators should be incentivised to link fleet size or permit renewals to the achievement of equity metrics.
- Comprehensive requirements: governance frameworks should adopt a multi-dimensional approach, including reduced fares, accessible payment systems, and targeted outreach.

On the other hand, there is a notable lack of studies addressing adapted business models to support the implementation of the 15-min city (15mC), particularly in the outskirts. It remains unclear how the principles of proximity can be transferred to low-and mid-density neighbourhoods, where the prevailing business models behind key services (such as public transport, shared mobility, and micromobility) are financially unsustainable due to lower demand compared to dense urban cores. This calls for the design of new, more resilient or socially-driven business models tailored to the specific conditions of peripheral areas.

Similar to the governance literature, research exploring business models within the 15mC framework is scarce. When it comes to the outskirts, there is virtually no study addressing this issue. Making services financially viable in these areas, in a way that enhances access to local activities, remains an underdeveloped research topic. The limited existing literature tends to focus on incentivising citizen engagement and participatory budgeting, or on broader strategies such as the creation of Special Economic Zones (SEZs), as suggested by Allam, Bibri, Chabaud et al. [1], which aim to attract investment, foster public–private collaboration, and stimulate local job creation.

Traditionally, public transport and the first station-based bike-sharing systems were primarily funded through public subsidies. In contrast, emerging shared mobility services—like micromobility and car-sharing—are often driven by private initiatives, making them less likely to operate in low-income or low-density neighbourhoods without public support [17, 49, 87]. Allam et al. [2] also advocate for the institutionalisation of fiscal incentives to align private sector investment with public goals. To develop resilient business models for the outskirts, stable and long-term funding strategies are essential. In this regard, leveraging the support of national and international programmes (such as the European Commission’s Climate-Neutral and Smart Cities Mission and the Circular Cities and Regions Initiative) could be key. In sum, resilient business models for the 15mC remains an overlooked yet critical gap, especially in peripheral contexts.

To clarify how governance structures and business models can support the implementation of the 15mC, a recent study by Lamíquiz-Daudén et al. [52] analysed the policies related to the proximity city in five Spanish cities (Barcelona, Castelló de la Plana, Pontevedra, Valladolid and Vitoria-Gasteiz) in order to identify how they have incorporated proximity planning in their plans. For instance, in Barcelona, the Superblocks program exemplifies a decentralised, municipally-led governance model that coordinates across urban planning, mobility, and environmental departments to reclaim street space for pedestrians and local uses. Vienna demonstrates how a public–private governance approach—particularly through its robust social housing system—can integrate proximity-based planning with long-term affordability and accessibility goals. In terms of business models, MaaS platforms illustrate how digital integration and public–private collaboration can enable flexible, multimodal travel options via subscription-based or pay-per-use systems. Moreover, policy initiatives such as Paris’s “Ville du Quart d’Heure,” Melbourne’s “20-Minute Neighbourhood” framework, and Portland’s Climate Action Plan showcase how cities have institutionalised proximity principles through zoning reforms, cross-sectoral policy alignment, and strategic infrastructure investments. These cases demonstrate that successful implementation requires not only physical design but also supportive institutional frameworks and adaptive governance mechanisms.

## Discussion

Our review reveals that while the 15mC concept is gaining traction, academic research remains heavily focused on its spatial elements: density, diversity, and design. These well-established physical elements form the backbone of proximity-based urban planning. However, this emphasis alone is insufficient to realise the 15mC’s potential—particularly in low- and mid-density contexts such as the urban outskirts. Our findings highlight the importance of three additional, yet often overlooked, elements that act as key enablers and that are equally critical for effective and inclusive 15mC implementation: individual’s characteristics and needs, digitalisation, and adapted governance and business models.

Together, these six enablers—spatial, social, digital, and institutional—should be understood as mutually reinforcing. For instance, digitalisation can expand access to services where density is low, while governance frameworks and funding models can ensure equity in service provision even when the private sector has limited incentive to operate. Similarly, proximity without attention to affordability, gender, age, or mobility constraints risks excluding vulnerable groups and deepening spatial inequalities. The outskirts pose particular challenges—due to dispersed built environments, weaker infrastructure, and lower service viability—that require a more integrated and tailored planning approach. Despite their importance, these three overlooked elements remain underdeveloped in the literature. In particular, the role of business models is strikingly absent, especially in suburban and peri-urban contexts. Yet these models are vital for sustaining services such as shared mobility, healthcare access, or local economic activity, without which the 15mC risks becoming a city-centre-centric ideal. Similarly, governance strategies—including participatory planning and equity-based regulation—have only recently begun to receive academic attention, and most studies stop short of offering actionable frameworks or metrics for implementation.

In light of these findings, we extend an invitation to rethink the 15mC concept, and move beyond the current rigid interpretation of it—typically focused on high-density urban cores—and to embrace a more flexible, context-sensitive perspective: the 15-min neighbourhood (15mN). From this context-adapted interpretation of the initial 15mC, 15-min neighbourhoods aim to reduce the need for private car use in the urban outskirts by allowing residents to access essential amenities and fulfil daily needs through active mobility and local infrastructure (such as public transport, shared mobility services, and mobility hubs), while remaining well-connected to other urban cores through regional transit systems. The 15-min threshold here should be interpreted not as a rigid rule but as a flexible reference point for promoting proximity-centred accessibility adapted to different spatial and social contexts.

Building upon Cervero’s 3D (later called 6D’s) [27] and Moreno’s 15mC four pillars [66], 15mN shifts the focus to the local scale, especially in urban outskirts, where achieving proximity requires different tools and strategies. In doing so, we reframe and point to crucial elements that need to be considered beside density, diversity, and design, including also individual characteristics and needs, digitalisation, and adapted governance and business models. Together, these six elements or key enablers, form a comprehensive foundation for future research and policy aimed at realising inclusive and sustainable proximity-based urbanism in diverse spatial contexts (see Table 3).

**Table 3:** Transitioning from the 15mC to the 15-min neighbourhood (15mN)

	Component	Sub−component	Description	Use
1	Density	Population density	Residential density of the urbanised area of a particular neighbourhood	Helping planners to identify the scale (city or neighbourhood level) of the 15mN policy/practice
2	Diversity	Amenities	Optimal (minimum) set of amenities that fit the specific neighbourhood’s needs for a certain time threshold	Determining which amenities are offered as essential services to have covered
3	Design	Built environment	Physical aspects	Characterising the neighbourhood in terms of public space quality (sidewalk width, cycling infrastructure, shaded paths, benches, greenery, etc.)
		Connectivity	Mobility Network	Characterising the neighbourhood in terms of local and regional transport options/modes available and its related infrastructure
4	Individual's characteristics and needs	Socioeconomic characteristics	Socioeconomic variables (gender, age, income, occupation, etc.)	Defining profiles that help target specific populations
		Needs and preferences	The needs and desires that people express (perceptions, opinions, wishes)	Designing policies/measures/solutions that respond to actual needs and desires
5	Digitalisation	Digital tools	Use of digital technologies such as e−governance platforms, Mobility−as−a−Service (MaaS), real−time data systems, digital public services, and telework/telehealth tools	Enhancing accessibility, participation, and service delivery—especially in low−density areas—by reducing reliance on physical proximity and enabling data−informed planning
6	Governance and business models	Policy	Planning documents with guidelines and measurable policy benchmarks tied to statutory weight aiming for proximity goals (e.g. Master/Regional Plans)	Strengthening planners’ ability to commit to the 15mC concept with a clear understanding of what is expected to be delivered
		Citizen participation	Collaboration in activities of any kind (surveys, workshops, focus groups, etc.) aiming to engage citizens in the design and implementation of their 15 −minute neighbourhoods	Increasing the acceptance and feasibility of policies because they have been built from a bottom−up approach obtaining the support of citizens
		Equity−oriented regulation	Requirements that the Municipalities and authorities can set for mobility providers to comply with equity targets (affordability, inclusivity and geographic distribution to unserved areas)	Helping planners and authorities to reach equity goals faster and aligned with the current services being offered
		Business models	Financial mechanisms that help mobility services become economically sustainable	Identifying the aspects that need to be considered for building resilient business models for mobility solutions in the outskirts

Source: own elaboration

As a limitation, we acknowledge that this review focused exclusively on peer-reviewed academic literature, thereby excluding potentially insights from grey literature and non-academic sources. While we recognise the importance of such literature in broadening perspectives, we deliberately chose to concentrate on peer-reviewed studies to ensure methodological transparency and objectivity. We found that there was a relatively sufficient number of academic work to conduct an evidence-based synthesis of vetted research, which we consider the most appropriate foundation for critical analysis at this stage. Nonetheless, future studies could expand the scope to include grey literature and policy reports to provide a more comprehensive and practice-oriented understanding of the topic.

## Conclusion

This paper presented a systematic literature review to examine how the 15-min city (15mC) concept can be meaningfully applied to non-central urban areas, particularly urban outskirts, which tend to be car-dependent and maintain strong functional and economic links to city centres. Our review identifies six critical elements for enabling the 15mC: three well-established in the literature—density, diversity, and design—and three less developed but equally essential: individual characteristics and needs, digitalisation, and adapted governance and business models.

Several important research gaps emerged from the reviewed studies. First, user perceptions and needs, particularly among specific population groups, are rarely addressed, limiting understanding of how proximity is experienced differently across socioeconomic profiles. Second, although walking and, to a lesser extent, cycling are often analysed, public transport access—especially in low-density contexts—is underexplored. Third, urban peripheries remain an understudied spatial category, despite their growing relevance for proximity-based planning. Furthermore, we found a lack of empirical studies on equity-driven governance mechanisms and sustainable business models that can support inclusive and viable mobility services in suburban areas. These gaps, concentrated in the social, digital, and institutional dimensions, represent structural limitations of the current 15mC paradigm.

To address these limitations, we invite readers to approach the urban outskirts through the lens of the 15-min neighbourhood (15mN)—a more flexible and context-sensitive framework suitable for the outskirts. This lens helps shift attention from rigid spatial thresholds toward holistic, locally adapted solutions that integrate spatial, social, and systemic enablers. In particular, we encourage future research to further investigate the role of digitalisation (its spatial and social implications), as it can significantly enhance accessibility in the outskirts, where low density and distance from services would otherwise challenge the 15-min threshold. Likewise, new resilient business models are needed to make shared and micromobility services viable in areas with lower demand, ensuring both economic and social sustainability.

Our literature review can help policy planners to balance the elements necessary to successfully implement the 15-min neighbourhoods within the urban outskirts. Each component works on a spectrum, reflecting different aspects, intensities and qualities of urban development. It illustrates that a 15-min neighbourhood is one achieved through careful adjustments and coordination across these multiple elements towards building a unified living environment that is sustainable and functional. As main long-term policy recommendations, we can highlight two: (1) the importance of recognising each area's unique context and (2) the need to incorporate the three currently overlooked elements—social needs, digital tools, and institutional mechanisms—into proximity-based planning.

In short, future research must move beyond the spatial paradigm to address the systemic enablers of proximity-based planning. The 15mC cannot be fully realised in the urban outskirts without considering who the users are, how their access needs differ, what digital and physical infrastructures exist, and which governance and funding mechanisms are in place to support lasting change. All six elements or key enablers identified in this review deserve equal analytical and practical attention. Addressing them holistically can help transform the 15mC from an abstract ideal into a workable framework for inclusive, sustainable, and context-responsive urban transformation.

## Supplementary Information

Additional file 1.
