# Supplementary material for: Exploring the 15-Minute City concept for the urban outskirts: a systematic literature review
Source: Eur Transp Res Rev. 2025 Oct 17;17(1):50. doi: 10.1186/s12544-025-00743-8 (PMC12534276; doi:10.1186/s12544-025-00743-8)
Supplement: Supplementary file 1 — Additional file 1. [file 12544_2025_743_MOESM1_ESM.docx]

**Supplementary material**

Appendix 1 Table of authors for general literature (48 studies) ordered and indexed by date of publication.

| **Study ID** | **Title** | **Year** | **Authors** |
| --- | --- | --- | --- |
| 1 | Residents’ preferences for walkable neighbourhoods | 2017 | (Brookfield, 2017) |
| 2 | How much is enough? Assessing the influence of neighbourhood walkability on undertaking 10-minute walks | 2018 | (Boisjoly et al., 2018) |
| 3 | 15-Minute City: Decomposing the New Urban Planning Eutopia | 2021 | (Pozoukidou & Chatziyiannaki, 2021) |
| 4 | A city of cities: Measuring how 15-minutes urban accessibility shapes human mobility in Barcelona | 2021 | (Graells-Garrido et al., 2021) |
| 5 | Introducing the “15-Minute City”: Sustainability, Resilience and Place Identity in Future Post-Pandemic Cities | 2021 | (Moreno et al., 2021) |
| 6 | 15-, 10- or 5-minute city? A focus on accessibility to services in Turin, Italy | 2022 | (Staricco, 2022) |
| 7 | A Grammar-Based Optimization Approach for Designing Urban Fabrics and Locating Amenities for 15-Minute Cities | 2022 | (Lima et al., 2022) |
| 8 | Barcelona under the 15-Minute City Lens: Mapping the Accessibility and Proximity Potential Based on Pedestrian Travel Times | 2022 | (Ferrer-ortiz et al., 2022) |
| 9 | Circle of paradigms? Or ‘15-minute’ neighbourhoods from the 1950s | 2022 | (Kissfazekas, 2022) |
| 10 | Exploring the 15-minute neighbourhoods. An evaluation based on the walkability performance to public facilities | 2022 | (Caselli et al., 2022) |
| 11 | Geospatial analysis framework for evaluating urban design typologies in relation with the 15-minute city standards | 2022 | (Burke et al., 2022) |
| 12 | is the 15-minute city within reach? Evaluating walking and cycling accessibility to grocery stores in Vancouver | 2022 | (Hosford et al., 2022) |
| 13 | Planning Innovation or City Branding? Exploring How Cities Operationalise the 20-Minute Neighbourhood Concept | 2022 | (Gower & Grodach, 2022) |
| 14 | The 15-minute city: interpreting the model to bring out urban resiliencies | 2022 | (Abdelfattah et al., 2022) |
| 15 | The 15-Minute City—The Geographical Proximity of Services in Krakow | 2022 | (Noworól et al., 2022) |
| 16 | The Level of Inclusiveness of Current 15-Minute City Models. A Qualitative Analysis on How Far City of Proximity Strategies and Design for All Are Merging | 2022 | (Ramírez Saiz et al., 2022) |
| 17 | Unpacking the '15-Minute City' via 6G, IoT, and Digital Twins: Towards a New Narrative for Increasing Urban Efficiency, Resilience, and Sustainability | 2022 | (Allam, Bibri, Jones, et al., 2022) |
| 18 | Urban mobility evolution and the 15-minute city model: from holistic to bottom-up approach | 2022 | (Papas et al., 2022) |
| 19 | Urban Planning in the 15-Minute City: Revisited under Sustainable and Smart City Developments until 2030 | 2022 | (Pozoukidou & Angelidou, 2022) |
| 20 | Scenarios for a Post-Pandemic City: urban planning strategies and challenges of making “Milan 15-minutes city” | 2022 | (Pinto & Akhavan, 2022) |
| 21 | The ‘15-Minute City’ concept can shape a net-zero urban future | 2022 | (Allam, Bibri, Chabaud, et al., 2022a) |
| 22 | Urban accessibility in a 15-minute city: a measure in the city of Naples, Italy | 2022 | (Gaglione et al., 2022) |
| 23 | Improving the Spatial Accessibility of Community-Level Healthcare Service toward the ‘15-Minute City’ Goal in China | 2022 | (Song et al., 2022) |
| 24 | A civic and sustainable 15-minute campus? Universities should embrace the 15-minute city concept to help create vibrant sustainable communities | 2022 | (Barratt & Swetnam, 2022) |
| 25 | 15-Minute City: Utopia or reality? | 2023 | (De Leániz & Lobo, 2023) |
| 26 | A composite X-minute city cycling accessibility metric and its role in assessing spatial and socioeconomic inequalities –A case study in Utrecht, the Netherlands | 2023 | (Knap et al., 2023) |
| 27 | Are Italian cities already 15-minute? Presenting the Next Proximity Index: A novel and scalable way to measure it, based on open data | 2023 | (Olivari et al., 2023) |
| 28 | Assessing spatial equity in urban park accessibility: an improve two-step catchment area method from the perspective of 15-mintue city concept | 2023 | (Zhang et al., 2023) |
| 29 | Community gardens and the 15-minute city: Scenario analysis of garden access in New York City | 2023 | (Limerick et al., 2023) |
| 30 | From lockdown to precise prevention: Adjusting epidemic-related spatial regulations from the perspectives of the 15-minute city and spatiotemporal planning | 2023 | (Y. Li et al., 2023) |
| 31 | Graph Representation of the 15-Minute City: A Comparison between Rome, London, and Paris | 2023 | (Barbieri et al., 2023) |
| 32 | Materials for a debate on the 15-minute city: Public transportation’s effect on urban space and time in two Asia-based alternative | 2023 | (Bruno et al., 2023) |
| 33 | Measuring compliance with the 15-minute city concept: State-of-the-art, major components and further requirements | 2023 | (Papadopoulos et al., 2023) |
| 34 | Rethinking urban utopianism: The fallacy of social mix in the 15-minute city | 2023 | (Casarin et al., 2023) |
| 35 | Spatial regression model of urban walkability under the 15-minute city approach | 2023 | (Aristizábal et al., 2023) |
| 36 | The 15-minute city for all? – Measuring individual and temporal variations in walking accessibility | 2023 | (Willberg et al., 2023) |
| 37 | The 15-minute city: Urban planning and design efforts toward creating sustainable neighbourhoods | 2023 | (Khavarian-Garmsir, Sharifi, & Sadeghi, 2023) |
| 38 | The inclusive 15-minute city: Walkability analysis with sidewalk networks | 2023 | (Rhoads et al., 2023) |
| 39 | Towards an equity-centred model of sustainable mobility: Integrating inequality and segregation challenges in the green mobility transition | 2023 | (Tammaru et al., 2023) |
| 40 | From Garden City to 15-Minute City: A Historical Perspective and Critical Assessment | 2023 | (Khavarian-Garmsir, Sharifi, Hajian Hossein Abadi, et al., 2023) |
| 41 | “15-Minute City” and Elderly People: Thinking about Healthy Cities | 2023 | (Ulloa-Leon et al., 2023) |
| 42 | The 15-minute city model: the case of Sicily during and after covid-19 | 2023 | (Basbas et al., 2023) |
| 43 | Mosques pedestrian accessibility analysis under the 15 min cities concept | 2023 | (Harroucha & Chaouni, 2023) |
| 44 | Developing the 15-Minute City: A comprehensive assessment of the status in Hong Kong | 2024 | (Liu, Kwan, & Wang, 2024) |
| 45 | Development of a Chrono-Urbanism Status Composite Index under the 5/10/15-Minute City Concept Using Social Media Big Data | 2024 | (Liu, Kwan, Wang, et al., 2024) |
| 46 | Do informal urban communities in Ghana qualify as a 15-Minute City based on access to socioeconomic activities? | 2024 | (Dumedah et al., 2024) |
| 47 | Reconceptualizing Proximity Measurement Approaches through the Urban Discourse on the X-Minute City | 2024 | (Megahed et al., 2024) |
| 48 | Spatial Equity of Urban Park Distribution: Examining the Floating Population within Urban Park Catchment Areas in the Context of the 15-Minute City | 2024 | (Jeon & Jung, 2024) |

Source: own elaboration.

Appendix 2 Table of authors for the outskirts literature (26 studies) ordered and indexed by date of publication.

| **Study ID** | **Title** | **Year** | **Authors** |
| --- | --- | --- | --- |
| 49 | Accessibility in Practice: 20-Minute City as a Sustainability Planning Goal | 2020 | (Da Silva et al., 2020) |
| 50 | 15-minute neighbourhood accessibility: A comparison between Naples and London | 2021 | (Gaglione et al., 2021) |
| 51 | Assessing Urban Accessibility in Monterrey, Mexico: A Transferable Approach to Evaluate Access to Main Destinations at the Metropolitan and Local Levels | 2021 | (Gaxiola-Beltrán et al., 2021) |
| 52 | COVID-19, activity and mobility patterns in Bogota. Are we ready for a ‘15-minute city’? | 2021 | (Guzman et al., 2021) |
| 53 | The Theoretical, Practical, and Technological Foundations of the 15 Minute City Model: Proximity and Its Environmental, Social and Economic Beneﬁts for Sustainability | 2022 | (Allam, Bibri, Chabaud, et al., 2022b) |
| 54 | The x-minute city: Measuring the 10, 15, 20-minute city and an evaluation of its use for sustainable urban design | 2022 | (Logan et al., 2022) |
| 55 | Urban Transition and the Return of Neighbourhood Planning. Questioning the Proximity Syndrome and the 15-Minute City | 2022 | (Marchigiani & Bonfantini, 2022) |
| 56 | A Case Study of a 15-Minute City Concept in Singapore’s 2040 Land Transport Master Plan: 20-Minute Towns and a 45-Minute City | 2022 | (Renaningtyas Manifesty & Park, 2022) |
| 57 | Achieving ‘Active’ 30 Minute Cities: How Feasible Is It to Reach Work within 30 Minutes Using Active Transport Modes? | 2022 | (Both et al., 2022) |
| 58 | Local Living and Travel Time based Urbanism | 2022 | (Gilbert & Woodcock, 2022) |
| 59 | The 20-minute city: An equity analysis of Liverpool City Region | 2022 | (Calafiore et al., 2022) |
| 60 | Trends and inequalities in distance to and use of nearest natural space in the context of the 20-min neighbourhood: A 4-wave national repeat cross-sectional study, 2013 to 2019 | 2022 | (Olsen et al., 2022) |
| 61 | Accessibility inequality across Europe: a comparison of 15-minute pedestrian accessibility in cities with 100,000 or more inhabitants | 2023 | (Vale & Lopes, 2023) |
| 62 | Micromobility in Urban Trail Paths: Expanding and Strengthening the Planning of 15-Minute Cities | 2023 | (Vizmpa et al., 2023) |
| 63 | Moving the 15-minute city beyond the urban core: The role of accessibility and public transport in the Netherlands | 2023 | (Poorthuis & Zook, 2023) |
| 64 | Quantifying and visualizing the 15-Minute walkable city concept across Europe: a multicriteria approach | 2023 | (Bartzokas-Tsiompras & Bakogiannis, 2023) |
| 65 | The 15-minute city concept and new working spaces: a planning perspective from Oslo and Lisbon | 2023 | (Di Marino et al., 2023) |
| 66 | Who is living a local lifestyle? Towards a better understanding of the 15-minute-city and 30-minute-city concepts from a behavioural perspective in Montréal, Canada | 2023 | (Birkenfeld et al., 2023) |
| 67 | Filling in the Spaces: Compactifying Cities towards Accessibility and Active Transport | 2023 | (Monteiro et al., 2023) |
| 68 | Availability and Adequacy of Facilities in 15 Minute Community Life Circle Located in Old and New Communities | 2023 | (Wu & Divigalpitiya, 2023) |
| 69 | The “15-minutes station”: a case study to evaluate the pedestrian accessibility of railway transport in Southern Italy | 2023 | (Fazio et al., 2023) |
| 70 | The Potential Role of Railway Stations and Public Transport Nodes in the Development of “15-Minute Cities” | 2023 | (Wolański, 2023) |
| 71 | Is proximity enough? A critical analysis of a 15-minute city considering individual perceptions | 2024 | (Guzman et al., 2024) |
| 72 | Developing a 15-minute city: A comparative study of four Italian Cities-Cagliari, Perugia, Pisa, and Trieste | 2024 | (Murgante, Patimisco, et al., 2024) |
| 73 | Developing a 15-minute city: Evaluating urban quality using configurational analysis. The case study of Terni and Matera, Italy | 2024 | (Murgante, Valluzzi, et al., 2024) |
| 74 | Built environment and the evolution of the “15-minute city”: A 25-year longitudinal study of 200 Swedish cities | 2024 | (Elldér, 2024) |

Source: own elaboration.

Appendix 3 Origins of the 15mC concept


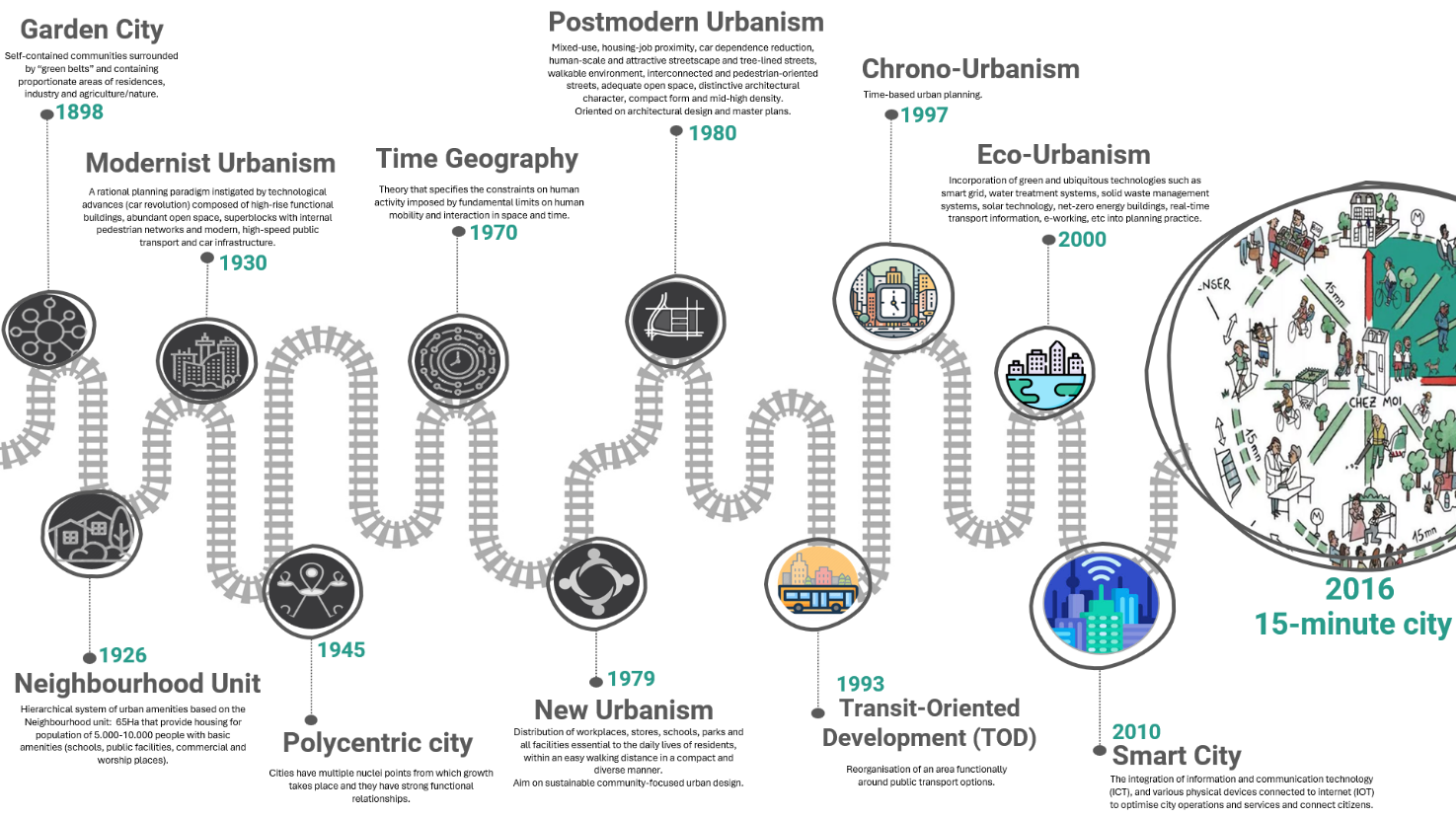


Source: own elaboration

Appendix 4 Overview of how authors handled amenities in their studies.

| **Study ID** | | **Spatial feature considered as origin** | | | | | | **Amenities** | | | | **Data Sources** | | | | | | | |  | |
| --- | --- | --- | --- | --- | --- | --- | --- | --- | --- | --- | --- | --- | --- | --- | --- | --- | --- | --- | --- | --- | --- |
|  |  | Grid-based | Census tracts | Postal code | Blocks/Cadastral parcels | Home address | Other | General Moreno | Weighted | Specific amenity | Other | Official sites (population, road network, etc.) | Mobility survey | OpenStreetMap (OSM) | Google Maps | GTFS | Other |  |  | |  |
|  |  |  |  |  |  |  |  |  |  |  |  |  |  |  |  |  |  |  | | |  |
|  |  |  |  |  |  |  |  |  |  |  |  |  |  |  |  |  |  |  | | |  |
| **General** | 4 |  | x |  |  |  |  |  | x (trip frequency) |  |  | x |  | x |  |  | x (Mobile Phone data) |  | | |  |
|  | 6 |  | x |  |  |  |  | x |  |  |  | x |  |  |  |  |  |  | | |  |
|  | 7 |  |  |  | x |  |  | x |  |  |  | x |  | x |  |  | x (Urban Grammar data) |  | | |  |
|  | 8 |  |  |  | x |  |  | x |  |  |  | x |  |  |  |  |  |  | | |  |
|  | 10 |  |  |  |  | x |  |  |  | x (Kindergartens) |  | x |  |  |  |  |  |  | | |  |
|  | 15 |  | x |  |  |  |  |  | x (trip frequency) |  |  | x |  | x |  |  |  |  | | |  |
|  | 22 |  | x |  |  |  |  | x |  |  |  | x |  |  | x |  |  |  | | |  |
|  | 26 | x |  |  |  |  |  |  | x (trip frequency) |  |  | x | x |  |  |  |  |  | | |  |
|  | 27 | x |  |  |  |  |  |  | x (trip frequency) |  |  |  |  | x |  |  |  |  | | |  |
|  | 31 |  |  |  | x |  |  | x |  |  |  |  |  | x |  |  |  |  | | |  |
|  | 35 |  |  |  |  |  | x (Traffic Analysis Zones- TAZ) | x |  |  |  | x |  |  |  |  |  |  | | |  |
|  | 42 |  |  |  |  |  | x (survey respondents) |  |  |  | x (survey respondents) |  |  |  |  |  | x (online survey) |  | | |  |
|  | 44 |  | x |  |  |  |  |  | x (# of check-ins) |  |  | x |  | x |  |  | x (check-ins from WEIBO app) |  | | |  |
| **Outskirts** | 49 |  |  |  | x |  |  | x |  |  |  | x |  |  |  |  |  |  | | |  |
|  | 50 |  | x |  |  |  |  | x |  |  |  |  |  |  | x |  |  |  | | |  |
|  | 51 |  |  |  | x |  |  | x |  |  |  | x |  | x |  |  |  |  | | |  |
|  | 52 |  |  |  |  |  | x (survey respondents) |  |  |  | x (survey respondents) |  |  |  |  |  | x (online survey) |  | | |  |
|  | 54 |  | x |  |  |  |  | x |  |  |  | x |  | x |  |  | x (OpenSourceRoutingMachine) |  | | |  |
|  | 57 |  | x |  |  |  |  |  |  | x (Work) |  | x |  | x |  | x |  |  | | |  |
|  | 62 |  |  |  |  |  | x (literature review study) |  |  |  | x (literature review study) |  |  |  |  |  | x (online scientific databases) |  | | |  |
|  | 63 |  |  | x |  |  |  | x |  |  |  | x | x |  |  | x | x (OpenTripPlanner) |  | | |  |
|  | 64 |  |  |  | x |  |  | x |  |  |  | x |  | x |  |  |  |  | | |  |
|  | 65 |  |  |  |  |  | x (Selection of working spaces) | x |  |  |  |  |  | x |  |  |  |  | | |  |
|  | 66 |  |  |  |  | x |  | x |  |  |  | x | x | x |  | x |  |  | | |  |
|  | 67 |  |  |  | x |  |  |  | x (trip frequency) |  |  | x | x |  |  |  | x (City's Master Plan) |  | | |  |
|  | 68 |  | x |  |  |  |  | x |  |  |  | x |  |  |  |  | x (BAIDU app) |  | | |  |
|  | 69 |  |  |  |  |  | x (selection of railway stations) | x |  |  |  |  |  | x |  |  |  |  | | |  |
|  | 72 |  | x |  |  |  |  |  | x (Analytic Hierarchy Process) |  |  | x |  | x | x |  |  |  | | |  |
|  | 73 |  | x |  |  |  |  |  | x (Analytic Hierarchy Process) |  |  | x |  | x | x |  |  |  | | |  |

Source: own elaboration.

Appendix 5 Socio-economic variables considered din the studies revised

| Study ID | | Age | Income | Gender | Education  level | Migrant Background | Other variables |
| --- | --- | --- | --- | --- | --- | --- | --- |
| Urban | 4 | x |  | x |  | x | x (Human Development Index) |
|  | 6 |  |  |  |  |  |  |
|  | 7 |  |  |  |  |  |  |
|  | 8 |  |  |  |  |  |  |
|  | 10 |  |  |  |  |  |  |
|  | 15 |  |  |  |  |  |  |
|  | 22 | x |  |  |  |  |  |
|  | 26 | x | x |  |  | x |  |
|  | 27 |  |  |  |  |  |  |
|  | 31 |  |  |  |  |  |  |
|  | 35 | x | x |  | x |  | x (veh ownership) |
|  | 42 | x |  | x | x |  |  |
|  | 44 |  | x |  |  |  |  |
| Outskirts | 49 |  |  |  |  |  | x (cyclists stress level) |
|  | 50 |  |  |  |  |  |  |
|  | 51 |  |  |  |  |  |  |
|  | 52 | x | x | x | x |  | x (occupation, veh ownership) |
|  | 54 |  |  |  |  |  |  |
|  | 57 |  |  |  |  |  | x (job types) |
|  | 62 |  |  |  |  |  |  |
|  | 63 | x | x |  |  | x | x (degree of urbanisation) |

Source: own elaboration

Appendix 6 Studies that conducted surveys to citizens about 15mC amenities.

| ID | Scope | Objective | Year and motivation | Type | Questions | Scale | Results |
| --- | --- | --- | --- | --- | --- | --- | --- |
| 71 | Full 15mC amenities preferences at detailed level | To developed an standardised index based on the availability of essential urban functions within a 15-minute walking distance. The index integrated individual preferences, along with geographical attributes, and the quality of pedestrian infrastructure. | Conducted in 2023  Motive: people prioritise essential services differently and within each type of services, specific amenities may also be valued differently. | Online  N=334 | First part: rank essential category: which is more important to have close to your home?  Second part: rank the 24 types of establishments.  - Imagine your own situation and neighbourhood...which category of service is more important to have close by? rank - Which specific services are more important in this category? rank  Third part: Sociodemographic and house location. | Urban  (Bogota, Colombia) | Grocery stores and most relevant across all populations. High-incomers had preference for retail shops and commercial services. Low-incomers for healthcare. |
| 52 | Not 15mC amenities preferences | To evaluate COVID-19 lockdown measures' impacts on activity and travel behaviours across different income groups. | Conducted in 2021 Motive: people changed their behaviours after the pandemic and perceptions of the lockdown measures may differ. | Online  N= 776 | First part: sociodemographic  Second part: travel decisions and time spent on specific activities (activity duration and possibility to perform online before, during and after COVID-19, trip mode, time and cost).  Third part: perceptual and qualitative information about government's measures (respondents asked to express their level of agreement with four statements related to time use satisfaction, financial concerns, agreement with the government mea­sures, and new technology adoption). | Urban  (Bogota, Colombia) | Low-income people were more exposed to contagion being forced to go out to find their daily sustenance. |
| 46 | Full 15mC amenities preferences at basic level | To evaluate the differences between perceived and actual travel times to work, healthcare, education, shopping, place of worship and family visit on a suburban context. | Conducted in 2021 Motive: perceived and realised accessibility is different. | Distributed forms  N=375 | First part: sociodemographic  Second part: travel times to different services, mode, cost and rank level of satisfaction with trip characteristics. | Rural- informal community  (Ayigya, Ghana) | Respondents declared (perception) longer travel times that the actual trip time (realised) as expected. However, it was demonstrated that in an informal area, the 15mC principles complied as most walked less than 5minutes to amenities. |
| 42 | Full 15mC amenities preferences at basic level | To evaluate the opinions of citizens with regards to the concept of 15-minute city | Conducted in 2020 and 2022 Motive: opinions about the usefulness of the 15mC concept might be different.  Biased sample: members of a social Facebook group of Sicilians who generally walk daily for both work and leisure. | Online  N=700 | First part: sociodemographic and car ownership Second part: do you consider walking as an anti-stress method? And what are the 8 most essential services for a neighbourhood to be within a 15 minute walking distance?  Third part: How useful do you think the concept of 15mC is for Sicilian cities? | 26% of sample from dense areas (300-700k pop).  44% of sample from mid-dense (urban outskirts) (60-300k pop.) 30% of sample from low-dense areas (less than 60k pop.) (Sicilian areas, Italy) | Residents thought the concept may be useful for Sicilian cities and 90% agreed that walking was an anti-stress method. Additionally, health services and green areas were ranked as the most important amenities. |
| Non | Full 15mC amenities preferences at detailed level | To investigate what citizens considered as "acceptable travel times" for different types of destinations, exploring the role of transport modes, and variations among different population groups. | Conducted in 2024 | Online  N= 3.468 | The relevance of reaching 16 different types of destinations.  Acceptable travel times to those destinations.  The current travel times to the nearest and most frequently visited locations.  The preferred modes of transportation for each destination.  Acceptable travel costs and other comfort-related factors | National (urban and outskirts) | 70% find access to supermarkets, healthcare (e.g., doctors, hospitals), and workplaces relevant. Education is also important for people with children. Supermarkets, daycare, metro stops 9-18min while work, university and hospital 30-50min. |

Source: own elaboration.

Appendix 7 Strategies recommended from the literature (governance and business models)

| Topic | Strategies | Infrastructure | Equity-based regulation | Business models | Information and  Citizen participation |
| --- | --- | --- | --- | --- | --- |
| Mobility | Expansion of shared mobility | x |  |  |  |
| Mobility | Improving public transport | x |  |  |  |
| Mobility | Improving pedestrian infrastructure | x |  |  |  |
| Mobility | Improving cycling infrastructure | x |  |  |  |
| Mobility | Creating mobility hubs | x |  |  |  |
| Housing | Unbundled parking for new house developments |  | x |  |  |
| Housing | Convert redundant office space into affordable housing |  | x |  |  |
| Housing | Mixed-income housing development |  | x |  |  |
| Housing | Introducing superblocks (pedestrianise areas) | x |  |  |  |
| Amenities | Increasing amenities (number and quality) | x |  |  |  |
| Amenities | Street experiments/activity hubs (taking space from cars) | x |  |  |  |
| Work | Offering flexible working schemes (co-working spaces) | x |  |  |  |
| Co-creation | Citizen participation with co-creation process (not just surveys but public meetings, walk-along interviews) |  |  |  | x |
| Technology | Augmented Reality to show possible interventions |  |  |  | x |
| Incentives | Incentives (monetary/non-monetary) to participate |  |  | x |  |
| Incentives | Fiscal incentives for private funds to align with public objectives |  |  | x |  |
| Policy | Outcome-oriented planning documents with benchmarks/indicators/statuary (e.g. everyone should have a hospital within a 30minutes PT ride) |  | x |  |  |
| Policy | Shift from 20-minute city to x-minute amenity (for example 30-minute hospital, 5-minute daycare, 10-minute bakery, 15-minute PT stop, etc.) |  | x |  |  |
| Regulation | Multi-purpose and multi-temporal use of facilities |  | x |  |  |
| Regulation | Mixed land use promotion |  | x |  |  |
| Regulation | Promote city planners trainings on how to get funds for projects |  | x |  |  |

Source: own elaboration

Appendix 8 13 additional studies included to cover relevant research during 2024-2025

| **Study ID** | **Title** | **Year** | **Authors** |
| --- | --- | --- | --- |
| 75 | The 15-minute city as paranoid urbanism: Ten critical reflections. | 2024 | (Caprotti et al., 2024) |
| 76 | Beyond the 15-minute city dichotomy: Time-denominated access to essential services in Chicago. | 2025 | (Ermagun et al., 2025) |
| 77 | Assessing the applicability of the 15-minute city: Insights from a spatial accessibility perspective. | 2025 | (Feng et al., 2025) |
| 78 | Proximity and Planning Tools in Spanish: the cases of Barcelona, Castelló de la Plana, Pontevedra, Valladolid and Vitoria-Gasteiz. | 2024 | (Lamíquiz-Daudén et al., 2024) |
| 79 | How workable is a 20-minute city in Australia? Re-examining the challenge of change in spatial labour and job relations. | 2025 | (T. Li et al., 2025) |
| 80 | Developing a 15-minute city policy? Understanding differences between policies and physical barriers. | 2025 | (Lu & Diab, 2025) |
| 81 | When proximity is not enough. A sociodemographic analysis of 15-minute city lifestyles. | 2025 | (Maciejewska et al., 2025) |
| 82 | Chrono-urbanism and liveable urban forms: A morphological analysis of alternative x-minute city models in Rosetta City. | 2025 | (Megahed et al., 2025) |
| 83 | Inclusive 15-minute cities: An age-sensitive assessment of active accessibility in the metropolitan area of Barcelona | 2025 | (Plaza-Herrera & Mercadé-Aloy, 2025) |
| 84 | How ’Smart’ is the 15-Minute City? Evaluating the Role of Technology in Advancing Accessibility, Mobility, and Well-being. | 2025 | (Popescu & Nicolescu, 2025) |
| 85 | X-minute cities as a growing notion of sustainable urbanism: A literature review. | 2025 | (Sepehri & Sharifi, 2025) |
| 86 | Towards completely caring 15-minute neighbourhoods. | 2025 | (Soukhov et al., 2025) |
| 87 | 15-minute city beyond the urban core: Lessons from the urban-suburban disparity in PCR accessibility within the X-minute framework. | 2025 | (Wang et al., 2025) |

Source: own elaboration.
